# Prg4 prevents osteoarthritis induced by dominant-negative interference of TGF-ß signaling in mice

**DOI:** 10.1371/journal.pone.0210601

**Published:** 2019-01-10

**Authors:** Robert Dalton Chavez, Philip Sohn, Rosa Serra

**Affiliations:** 1 Department of Biomedical Engineering, University of Alabama at Birmingham, Birmingham, Alabama, United States of America; 2 Department of Cell, Developmental, and Integrative Biology, University of Alabama at Birmingham, Birmingham, Alabama, United States of America; University of South Carolina, UNITED STATES

## Abstract

**Objective:**

Prg4, also known as Lubricin, acts as a joint/boundary lubricant. Prg4 has been used to prevent surgically induced osteoarthritis (OA) in mice. Surgically induced OA serves as a good model for post-traumatic OA but is not ideal for recapitulating age-related OA. Reduced expression of the TGF-β type II receptor (TGFβR2) is associated with age-related OA in clinical samples, so we previously characterized a mouse model that exhibits OA due to expression of a mutated dominant-negative form of TGFβR2 (DNIIR). Prg4 expression was significantly reduced in DNIIR mice. Furthermore, we showed that Prg4 was a transcriptional target of TGF-ß via activation of Smad3, the main signal transducing protein for TGF-ß. The objective of the present study was to determine whether maintenance of Prg4, a down-stream transcriptional target of TGF-ß, prevents OA associated with attenuated TGF-ß signaling in mice.

**Design:**

Wild-type, DNIIR, and bitransgenic mice that express both DNIIR and Prg4, were compared. Mice were assessed with a foot misplacement behavioral test, μCT, histology, and Western blot.

**Results:**

Compared to DNIIR mice, bitransgenic DNIIR+Prg4 mice missed 1.3 (0.4, 2.1) fewer steps while walking (mean difference (95% confidence interval)), exhibited a cartilage fibrillation score that was 1.8 (0.4, 3.1) points lower, exhibited cartilage that was 28.2 (0.5, 55.9) μm thicker, and exhibited an OARSI score that was 6.8 (-0.9, 14.5) points lower. However, maintenance of Prg4 expression did not restore levels of phosphorylated Smad3 in DNIIR mice, indicating Prg4 does not simply stimulate TGF-ß signaling.

**Conclusions:**

Our results indicate that maintenance of Prg4 expression prevents OA progression associated with reduced TGF-β signaling in mice. Since there was no evidence that Prg4 acts by stimulating the TGF-ß signaling cascade, we propose that Prg4, a transcriptional target of TGF-ß, attenuates OA progression through its joint lubrication function.

## Introduction

The molecular mechanisms that control age-related osteoarthritis (OA) are not well defined. Understanding the factors that maintain cartilage through aging may help identify targets that could be leveraged for prevention or treatment of age-related OA. The transforming growth factor beta (TGF-β) signaling pathway exhibits changes during age-related OA in humans. Specifically, human OA cartilage has been shown to exhibit reduced levels of the TGF-β type II receptor (TGFBR2) [1] and genetic variations in the SMAD3 gene are associated susceptibility to OA [2]. Also, in young, healthy chondrocytes, TGF-β mainly signals through the TGF-β type I receptor (Tgfbr1/ALK5) and Smad2/3, whereas in aged mice, the level of an alternate type I receptor, Acvrl1 (ALK1), which uses Smad1/5, is increased [3, 4]. It was suggested that signaling through Tgfbr1 provides a chondroprotective signal while signaling through Acvrl1 is catabolic. Investigation into proteins that mediate TGF-β’s chondroprotective effects may reveal useful information on the pathogenesis of age-related OA and how it could be prevented or treated. Chondroprotective targets of TGF-ß would be expected to have fewer side-effects than TGF-ß itself, which can have both positive and negative effects on cartilage that are often age dependent.

OA mouse models have been created by mimicking alterations to TGF-β signaling observed during the course of human OA. For example, we previously described a mouse model of OA created through expression of a dominant-negative mutant of TGFβR2 (DNIIR) [5, 6], while another mouse model was generated through disruption of the Smad3 gene (*Smad3*^ex8/ex8^) [7]. Specifically, the DNIIR and *Smad3*^ex8/ex8^ mouse models exhibited articular cartilage fibrillation, reduced articular cartilage thickness, and reduced proteoglycan staining [5–7]. Animal models like these may be able to improve understanding of the role of TGF-β and its mediators in age-related OA.

Proteoglycan 4 (PRG4), also known as Lubricin or Superficial Zone Protein, acts as a lubricant in joints [8]. For example, PRG4 isolated from human knees and recombinant human PRG4 both exhibit lubricating abilities [9, 10], and adding PRG4 to cartilage surfaces lowers the static coefficient of friction [11]. During OA in humans, PRG4 is down-regulated in lateral femoral condyle cartilage [12]. Prg4-overexpressing mice exhibit increased cartilage volume [13]. Prg4 knockout mice exhibit reduced proteoglycan staining and increased friction on cartilage surfaces [8]. PRG4 gene therapies have been tested in post-traumatic OA mouse models and have shown positive results [13, 14]. Nevertheless, Prg4 has not been tested in models of low TGF-β/ Smad2/3 signaling or in clinical trials for age-related OA.

Investigating molecular mechanisms that regulate Prg4 expression may result in useful pharmacological discoveries. TGF-β regulates PRG4 expression [6, 15–18]. Specifically, TGF-ß regulates PRG4 transcription through SMAD3 [15] and partially through the PKA-CREB signaling pathway [19]. CREB, along with Wnt signaling, PGE2, and an extracellular ATP signaling pathway, have been shown to mediate Prg4 expression under mechanical loading [20]. TGF-β and SMAD3 also regulate alternative splicing of *PRG4*, which alters the structure of the PRG4 molecule and affects how well it binds to extracellular matrix [16]. In addition, dominant-negative interference of TGF-ß signaling in mouse cartilage results in OA and down-regulation of *Prg4* mRNA expression, suggesting that Prg4 may mediate some of the chondroprotective effects of TGF-ß on cartilage [6]. Additional work on the relationship between TGF-β, Prg4, and chondroprotection is needed.

The goal of the present study was to test whether maintenance of Prg4 prevents development of OA in DNIIR mice. Maintenance of Prg4 in DNIIR mice was shown to prevent foot misplacement, cartilage fibrillation, cartilage thinning, loss of superficial zone cartilage, and development of pathologic OARSI scores. The primary new finding of this study is that maintenance of Prg4 expression attenuates the OA phenotype associated with interference of TGF-β signaling in mice. To determine if Prg4 acted to prevent OA in DNIIR mice by simply stimulating the TGF-ß signaling cascade, we assessed phosphorylated levels of Smad3 (pSmad3) [6, 15, 21]. Maintenance of Prg4 in DNIIR mice did not preserve pSmad3 levels suggesting Prg4 did not simply stimulate TGF-ß /Smad3 signaling. We thus propose that Prg4 attenuates OA in DNIIR mice through its joint lubrication function.

## Materials and methods

### Mice

DNIIR mice (MTR4 line) [5] and mice overexpressing Prg4 [13] were used. DNIIR mice express a cytoplasmically truncated, functionally inactive TGFβR2 under the control of a metallothionein-like promoter, which results in OA. Prg4 mice express Prg4 under the control of a Col2a1 promoter. Previously, DNIIR mice were in the C57BL/6 background, and Prg4 mice were in the FVB/N background. In the present study, all mice were generated from crosses of hemizygous DNIIR and Prg4 mice producing F1 offspring with a combined C57BL/6 and FVB/N background. Thus, all mice used in this study had the same genetic background. This breeding scheme produced four kinds of offspring: (1) wild-type mice with neither DNIIR nor Prg4 transgenes, (2) mice hemizygous for the DNIIR transgene (denoted as “*DNIIR*^Tg^”), (3) mice hemizygous for the Prg4 transgene (denoted as “*Prg4*^Tg^”), and (4) mice hemizygous for both DNIIR and Prg4 transgenes (denoted as “*DNIIR*^Tg^*;Prg4*^Tg^”). Mice were genotyped via PCR using DNA extracted from mouse tails. The primer sequences for genotyping were described previously [5, 13]. Mice were allocated into experimental groups based on genotype. Biological replicates are denoted with “n =“ in the figure legends; one biological replicate is defined as one mouse. Females and males were used. Mice were housed in a specific pathogen free facility in standard individually ventilated cages with bedding material. No more than 7 adult mice were housed per cage. A 12-hour light/dark cycle was used. Mice were kept at room temperature. Mice were fed a certified mouse diet of food and water *ad libitum*. Mice had environmental enrichment in the form of cardboard tubes and nesting material. The mouse protocol was approved by the Institutional Animal Care and Use Committee at the University of Alabama at Birmingham (protocol # IACUC-09811), and all animals were handled under the approved protocol.

### Western blots

Articular cartilage was harvested from joints of 18-month-old mice (for TGFβR2 blots) and 6-month-old mice (for pSmad3 and Smad2/3 blots). Five μg of protein lysate (for TGFβR2 blots) or 15 μg of protein lysate (for pSmad3 and Smad2/3 blots) per sample were separated by reducing electrophoresis on 4–20% polyacrylamide gels (Bio-Rad Laboratories, # 456–8096). Protein was transferred from gels to polyvinylidene fluoride membranes (Bio-Rad Laboratories, # 162–0177) using a Trans-Blot Turbo Transfer System (Bio-Rad Laboratories, # 1704150). Membranes were blocked with either 3% milk (Santa Cruz Biotechnology, # sc-2325) (for TGFβR2 blots) or 3% bovine serum albumin (Fisher Scientific, # BP1605-100) (for pSmad3 and Smad2/3 blots). Membranes were then incubated overnight with either anti-TGFβR2 primary antibody (1:1000, Abcam, # ab184948, raised in rabbit, monoclonal), anti-pSmad3 primary antibody (1:1000, Cell Signaling Technology, # 9520S, raised in rabbit, monoclonal), or anti-Smad2/3 primary antibody (1:1000, Cell Signaling Technology, # 8685S, raised in rabbit, monoclonal). Membranes were stripped between pSmad3 and Smad2/3 incubations. To assess whether equivalent amounts of protein were loaded in all wells, an anti-cyclophilin B primary antibody (1:1000, Abcam, # ab16045, raised in rabbit, polyclonal) was used. Membranes were washed with Tris-buffered saline containing 0.1% Tween 20 (TBST) and incubated with HRP-conjugated anti-rabbit (1:2000, Cell Signaling Technology, # 7074S, raised in goat) or anti-mouse (1:1000, Santa Cruz Biotechnology, # sc-2055, raised in goat) secondary antibodies. Images of Western blots were acquired on a ChemiDoc MP system (Bio-Rad Laboratories). Western blot quantification was performed with ImageJ (version 1.50i). For all Western blots, each group had n = 2–8 mice. For each mouse, protein was harvested from 2–4 hind limb joints (knees and hips) and pooled for that individual mouse. Approximately equal volumes of cartilage were harvested from the knees and hips.

### Immunohistochemistry and histology

For Prg4 immunohistochemistry, knee joints were harvested from 2-month-old mice. For all other histology, knee joints were harvested from 18-month-old mice. Knee joints were fixed in 4% paraformaldehyde overnight and placed in decalcification buffer on a rocker at 4°C for either 1 week (for 2-month-old samples) or 3 weeks (for 18-month-old samples). Samples were processed then embedded in paraffin. Sections were cut at a thickness of 6 μm and mounted on UltraClear slides (Denville, # M1021). For Prg4 immunohistochemistry, the following steps were taken. Antigen retrieval was performed by incubating sections in 0.02% proteinase K (Sigma Aldrich, # P2308) in mouse tail DNA isolation buffer at room temperature for 10 minutes. Sections were incubated overnight with anti-Prg4 primary antibody (1:200, Thermo Fisher Scientific, # PA3118), incubated for 1 hour with HRP-conjugated anti-rabbit secondary antibody (1:1000, Santa Cruz Biotechnology, # sc-2004), and incubated with 3,3’-diaminobenzidine (DAB) substrate. Methyl green was used as a counter stain. In addition to staining sections with both primary and secondary antibodies, separate sections were stained with only the secondary antibody as a control (S1 Fig). For all other histology, sections were stained with either hematoxylin and eosin (H&E) or Safranin O and fast green as described previously [22, 23]. For all histology, each group had n = 3–5 mice. For each mouse, one knee joint was harvested.

### Foot misplacement test

Mice underwent a foot misplacement behavioral test at 18 months of age. Mice were placed in the testing room for 30 minutes before testing. Mice were tested in the afternoon. An Automatic Foot Misplacement Apparatus (Bioseb In Vivo Research Instruments, # BIO-FMA) with a mouse corridor was used. The floor of the corridor has ladder rungs, and mice walk on the ladder rungs. Sensors identify the number of times the paws of the mice slip through the ladder rungs. Locotronic software (Bioseb In Vivo Research Instruments) was used to record the number of missed steps. Mice were placed at one end of the corridor and allowed to walk the length of the corridor. Mice were then allowed to walk back through the corridor to the original starting point. Missed steps on the return walk were not recorded. This back-and-forth walk was repeated 3 additional times for a total of 4 back-and-forth walks. The initial back-and-forth walk served as “practice” and was excluded from analysis. If a mouse changed direction midway through the recording, the walk was repeated until the mouse walked the full length of the corridor without changing directions. Three recordings per mouse were averaged to obtain a number of missed steps per mouse. Each group had n = 6–9 mice.

### μCT

Distal femurs were harvested from 18-month-old mice. In addition to control and bitransgenic groups, *DNIIR*^Tg^ mice were further divided by low and high DNIIR expression as determined by western blot analysis (S2 and S3 Figs). *DNIIR*^Tg^ mice with high DNIIR expression exhibited fibrillation that was detectable via μCT whereas low expressers did not present with fibrillation. As a result, our μCT evaluation focused on the subset of *DNIIR*^Tg^ mice with high DNIIR expression. Femurs were stored in PBS at 4°C until imaging. Imaging was performed within 48 hours of tissue harvest. μCT images of femoral articular cartilage were obtained using a protocol described previously that uses Hexabrix counterstain to visualize cartilage [24]. The protocol is summarized as follows. An imaging solution was prepared fresh on the day of use. The imaging solution consisted of 15% Hexabrix contrast agent (Guerbet, # 67684-5505-2) in PBS with protease inhibitors (Roche, # 05892970001). Each femur was placed in 1 mL of the imaging solution in a 1.5-mL Eppendorph tube. The tube was placed in a water bath at 37°C for 30 minutes. The femur was secured in a 12-mm-diameter μCT scanning holder. A μCT 40 desktop scanner (Scanco Medical) was used to image the samples in air at 45 kVp, 145 μA, and 300 ms integration time. The total scan time per sample was 63 minutes. Two-dimensional (2D) transverse slices were obtained. The μCT Evaluation Program (version 6.5–2, Scanco Medical) was used to manually select the cartilage regions in the 2D images. Images were thresholded with a low value of 553 and a high value of 737. For each sample, 212 2D slices were reconstructed into a three-dimensional (3D) image. Each group had n = 3–7 mice.

### Indentation test for thickness

Proximal tibiae were harvested from 18-month-old mice. Tibiae were stored in PBS at 4°C until tested. Testing was performed within 48 hours of tissue harvest. Articular cartilage on the lateral tibial plateau was tested for thickness using a protocol described previously [6]. The protocol is summarized as follows. A computer-controlled electromechanical test system (Bose LM1 ElectroForce TestBench) was fitted with a 250-g load cell (Bose). Tibiae were embedded in polymethylmethacrylate and immersed in PBS. The articular cartilage was kept wet during the embedding process. A sharp tungsten needle with a 1-μm tip radius was secured onto the load cell. The needle was pushed through the articular cartilage at a rate of 5 μm/s. Load-displacement curves were recorded, and cartilage thickness was determined from the curves as described previously [25]. Each group had n = 3–5 mice. For each mouse, one proximal tibia was analyzed.

### Statistical methods

Microsoft Excel (version 14.4.0) was used for all statistical tests unless otherwise noted. An Anderson–Darling test was performed to assess the data for normality. The *DNIIR*^Tg^*;Prg4*^Tg^ group in the foot misplacement data set, the majority of groups in the μCT data set, and the *DNIIR*^Tg^ group in the OARSI score data set were not normally distributed, but all other data was normally distributed. An f-test was performed to assess whether variances between groups were equal. In the foot misplacement and OARSI score data sets, the variance of the *DNIIR*^Tg^ group was different than the variance of the other groups, but the other groups had equal variance with each other. In all other data sets, all groups had equal variance with each other. For each data set, groups were analyzed via ANOVA using GraphPad Prism (version 6.0f). All ANOVA p-values were less than 0.05. Therefore, pairwise comparisons were performed. The Student’s t-test (two-tailed, unpaired) for equal variance was used for pairwise comparisons. A p-value of less than 0.05 was considered statistically significant. GraphPad Prism was used to calculate means, 95% confidence intervals, mean differences, and 95% confidence intervals for the mean differences. All graphs were made with GraphPad Prism.

## Results

### Prg4 prevents foot misplacement in *DNIIR*^Tg^ mice

**Fig 1:**
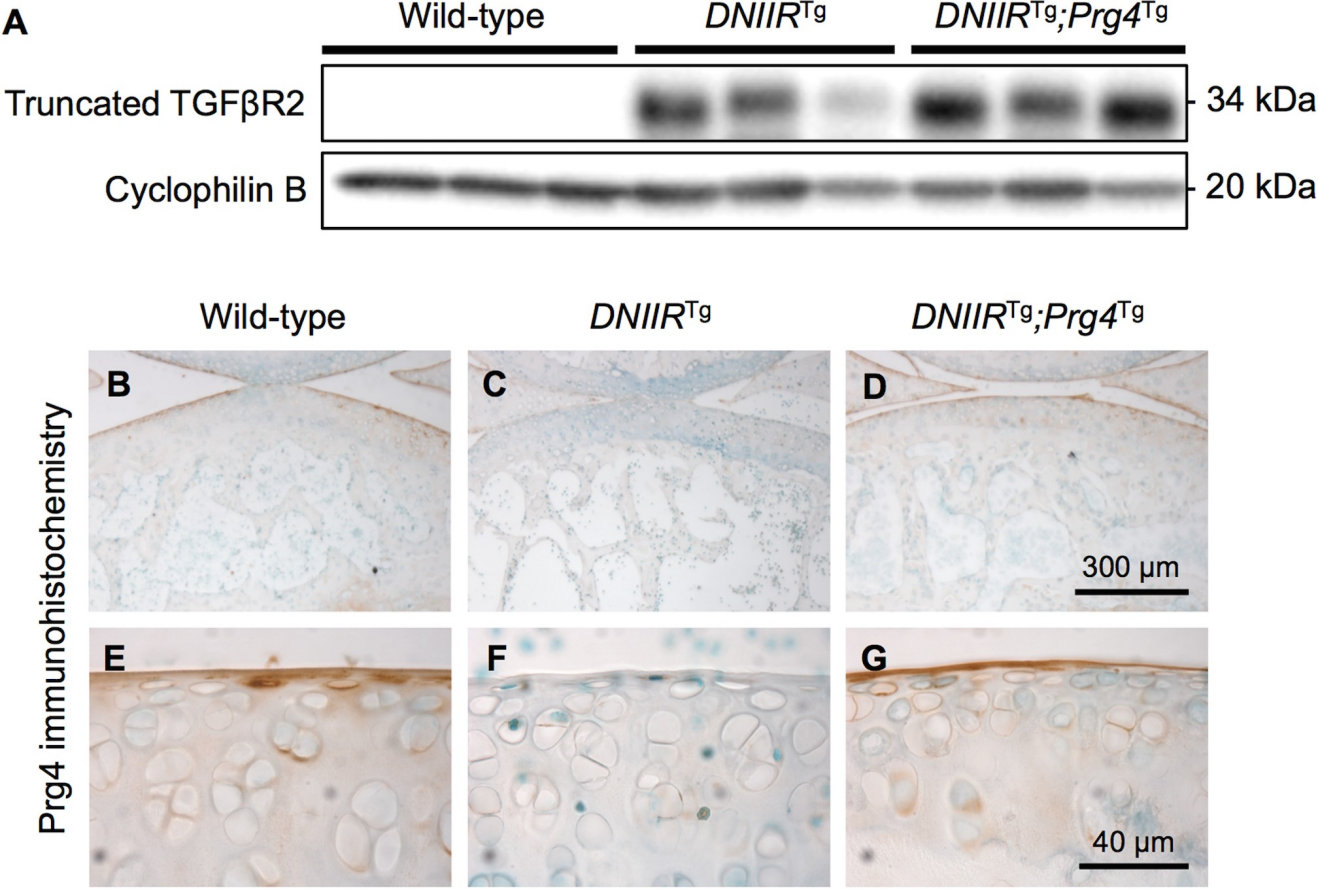
DNIIR and Prg4 expression. **(A)** Protein was collected from the articular cartilage of hind limb joints of 18-month-old mice. DNIIR protein levels were determined by Western blot. An antibody specific to the extracellular domain of TGFβR2 was used. A band corresponding to the truncated protein appeared at 34 kDa. Wild-type mice exhibited no DNIIR protein, while *DNIIR*^Tg^ and *DNIIR*^Tg^*;Prg4*^Tg^ mice exhibited a band representing the truncated TGFβR2 receptor. **(B-G)** Prg4 protein was identified via immunohistochemistry in 2-month-old mice. The articular cartilage of wild-type mice (B, E) exhibited Prg4 staining in the superficial zone and faint Prg4 staining in the middle zone. The articular cartilage of *DNIIR*^Tg^ mice (C, F) exhibited almost no Prg4 staining. The articular cartilage of *DNIIR*^Tg^*;Prg4*^Tg^ mice (D, G) exhibited staining in the superficial zone and faint staining in the middle zone, similar to the staining profile of the wild-type cartilage. Representative images are shown from wild-type mice (n = 3), *DNIIR*^Tg^ mice (n = 3), and *DNIIR*^Tg^*;Prg4*^Tg^ mice (n = 2).

Expression of DNIIR and Prg4 proteins was confirmed via Western blot and immunohistochemistry, respectively. Specifically, the articular cartilage of wild-type mice did not express DNIIR protein, while the articular cartilage of *DNIIR*^Tg^ and *DNIIR*^Tg^*;Prg4*^Tg^ mice expressed DNIIR protein (Fig 1A and S4 and S5 Figs). As expected, articular cartilage of *DNIIR*^Tg^ mice (Fig 1C and 1F) exhibited noticeably less Prg4 protein than the wild-type controls (Fig 1B and 1E). Prg4 expression was maintained in *DNIIR*^Tg^*;Prg4*^Tg^ mice (Fig 1D and 1G), which exhibited similar amounts of Prg4 protein to wild-type mice.

**Fig 2:**
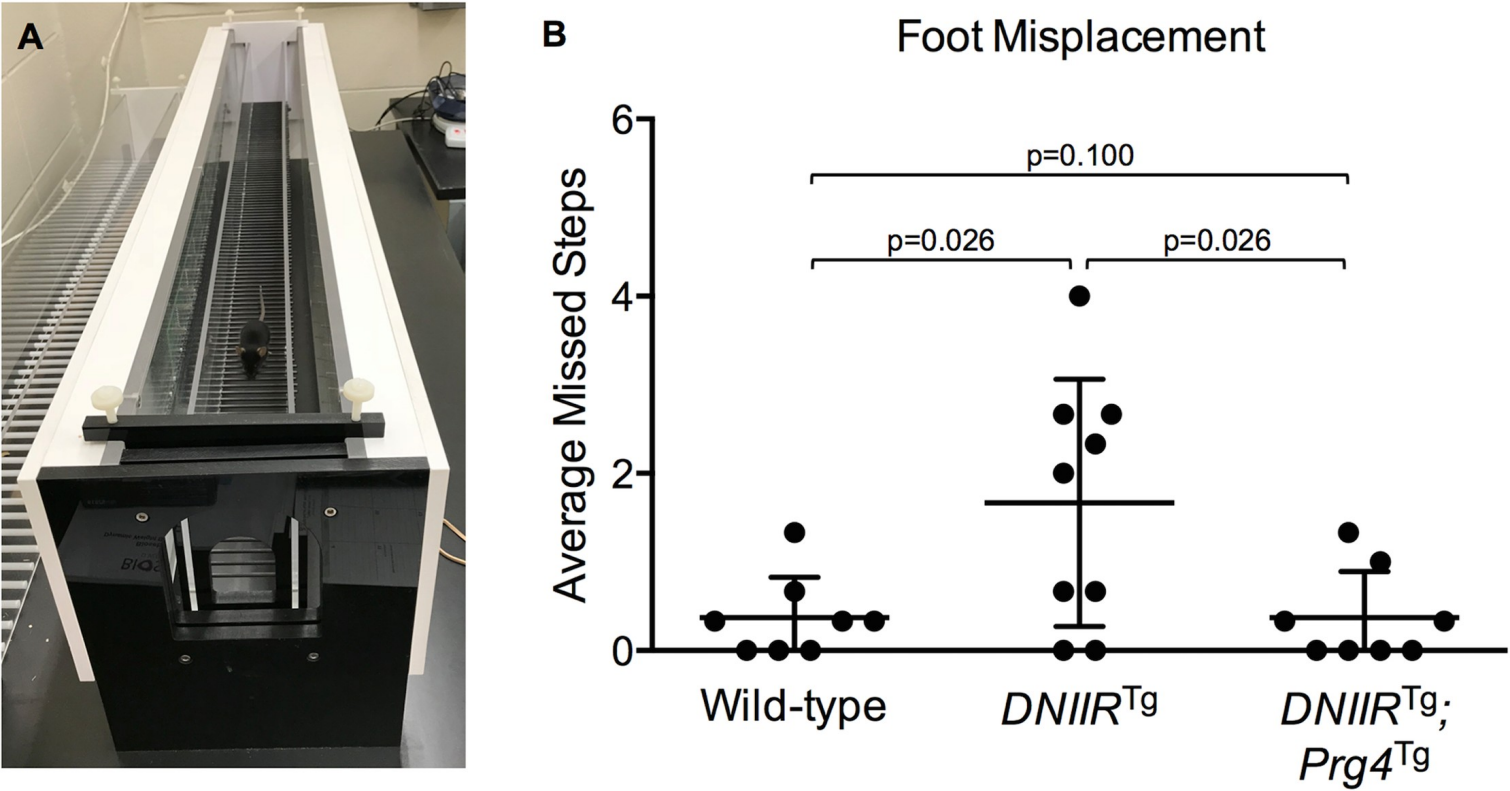
Prg4 prevents foot misplacement in *DNIIR*^Tg^ mice. (A) Mice at 18 months of age were placed in an apparatus that recorded the number of missed steps while walking. The following results are reported as “mean (lower bound of the confidence interval, upper bound of the confidence interval).” (B) Wild-type mice missed 0.4 (0.0, 0.8) steps (n = 8; 4 female, 4 male), *DNIIR*^Tg^ mice missed 1.7 (0.6, 2.7) steps (n = 9; 4 female, 5 male), and *DNIIR*^Tg^*;Prg4*^Tg^ mice missed 0.4 (-0.1, 0.8) steps (n = 8; 5 female, 3 male). There were the following statistically significant differences (reported as “mean difference (lower bound of confidence interval, upper bound of confidence interval)”): wild-type and *DNIIR*^Tg^ mice (-1.3 (-2.1, -0.4)) and *DNIIR*^Tg^ and *DNIIR*^Tg^*;Prg4*^Tg^ mice (1.3 (0.4, 2.1)).

Since patients with severe OA may stumble while walking [26], mouse walking behavior was assessed. Mice were aged to 18 months to mimic the stage of life of OA patients with altered TGF-β signaling [1] and because the *DNIIR*^Tg^ mice exhibited a noticeably more severe OA phenotype at 18 months of age compared to earlier ages. An apparatus was used to automatically record the number of steps that mice missed while walking (Fig 2A). An increase in missed steps was observed in *DNIIR*^Tg^ mice when compared to wild-type controls consistent with the presence of OA in these mice (Fig 2B). Foot misplacement was reduced to normal levels in the bitransgenic *DNIIR*^Tg^*;Prg4*^Tg^ mice, suggesting that Prg4 alleviated some of the consequences of OA in the DNIIR mice.

### Prg4 prevents cartilage fibrillation, cartilage thinning, and reduction of proteoglycan staining in *DNIIR*^Tg^ mice

**Fig 3:**
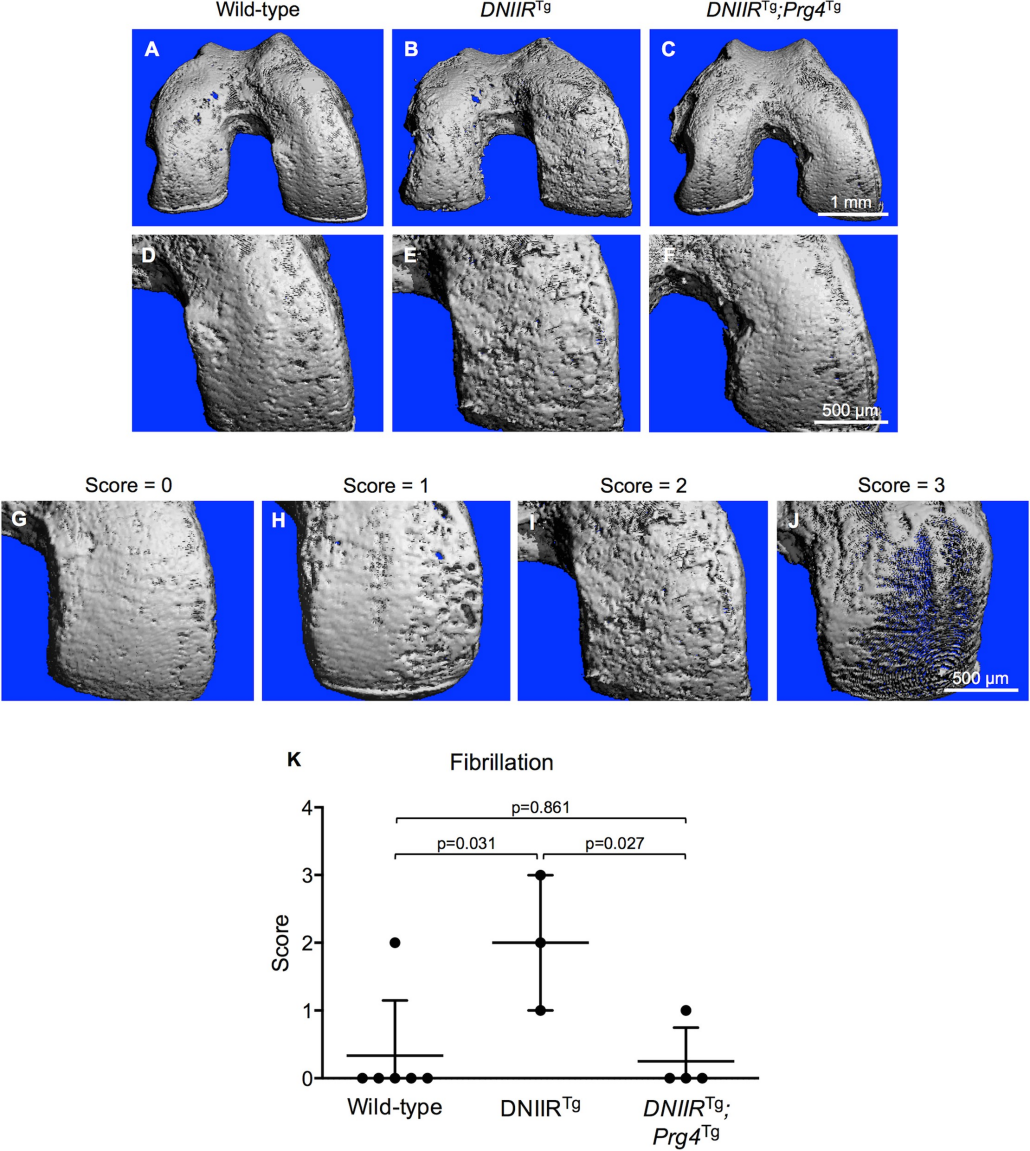
Prg4 prevents fibrillation of articular cartilage in *DNIIR*^Tg^ mice. μCT imaging was used to obtain 3D images of the surface of femoral articular cartilage in 18-month-old mice. (A-B, D-E) Wild-type mice exhibited mostly smooth cartilage, while *DNIIR*^Tg^ mice exhibited more fibrillation, especially in load-bearing regions. (C and F) *DNIIR*^Tg^*;Prg4*^Tg^ mice exhibited smoother cartilage than *DNIIR*^Tg^ mice. (D and F) Wild-type cartilage and *DNIIR*^Tg^*;Prg4*^Tg^ cartilage appeared similarly smooth. The medial condyle is shown up-close in D-F. To quantify the fibrillation on the medial condyle, we developed a scoring system. (G) A score of 0 corresponds to no fibrillation or very minimal fibrillation; (H) a score of 1 corresponds to fibrillation that partially covers the medial condyle; (I) a score of 2 corresponds to fibrillation that fully covers the medial condyle and consists of mostly shallow fissures; (J) and a score of 3 corresponds to fibrillation that fully covers the medial condyle and consists of mostly deep fissures. Images were scored by an observer blinded to genotype. The following results are reported as “mean (lower bound of the confidence interval, upper bound of the confidence interval).” (K) The fibrillation score was 0.3 (-0.5, 1.2) for wild-type mice (n = 6; 2 female, 4 male), 2.0 (-0.5, 4.5) for *DNIIR*^Tg^ mice (n = 3; 1 female, 2 male), and 0.3 (-0.5, 1.0) for *DNIIR*^Tg^*;Prg4*^Tg^ mice (n = 4; all 4 male). There were the following statistically significant differences (reported as “mean difference (lower bound of confidence interval, upper bound of confidence interval)”): wild-type and *DNIIR*^Tg^ mice (-1.7 (-2.9, -0.4)) and *DNIIR*^Tg^ and *DNIIR*^Tg^*;Prg4*^Tg^ mice (1.8 (0.4, 3.1)).

Cartilage fibrillation is a hallmark of OA in humans [27]. To look for fibrillation across the entire articular cartilage surface, we used a method of μCT imaging that can identify soft tissues and has been validated specifically for imaging articular cartilage [24]. As expected, high DNIIR-expressing mice exhibited more fibrillation when compared to wild-type control mice (Fig 3A, 3B, 3D, 3E and 3K). In human femurs, cartilage on the medial condyles experiences the largest stresses out of all regions across the distal femoral cartilage surface [28, 29], and this is also where fibrillation first appears [30]. In the present study, most fibrillation in *DNIIR*^Tg^ mice appeared on the medial condyle. To determine whether Prg4 could prevent fibrillation associated with high DNIIR expression, we looked at fibrillation in high DNIIR-expressing bitransgenic *DNIIR*^Tg^*;Prg4*^Tg^ mice. These mice exhibited minimal fibrillation (Fig 3C, 3F and 3K), similar to controls suggesting that Prg4 can prevent cartilage fibrillation that results from interference of TGF-ß signaling.

**Fig 4:**
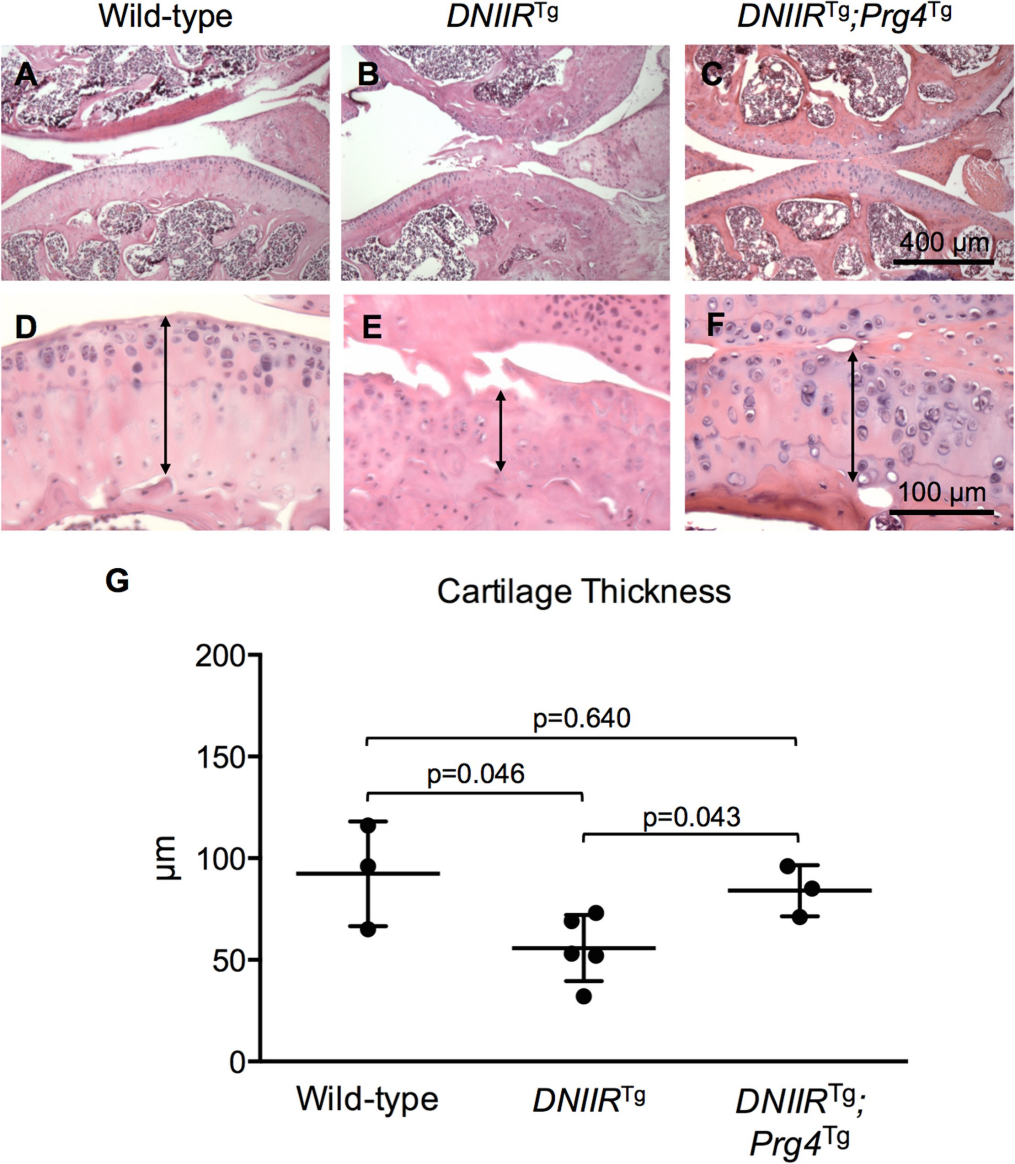
Prg4 prevents thinning of articular cartilage in *DNIIR*^Tg^ mice. H&E staining was used to visualize general histological characteristics of the articular cartilage in 18-month-old mice. The double arrows mark the thickness of the articular cartilage. (A-B, D-E) Wild-type tibial cartilage was thicker than *DNIIR*^Tg^ tibial cartilage. (C and F) *DNIIR*^Tg^*;Prg4*^Tg^ tibial cartilage was thicker than *DNIIR*^Tg^ tibial cartilage and similar in thickness to wild-type tibial cartilage. *DNIIR*^Tg^ cartilage exhibited fibrillation, whereas wild-type and *DNIIR*^Tg^*;Prg4*^Tg^ cartilage exhibited little, if any, fibrillation (n = 3 for each group). A needle indenter was used to confirm cartilage thickness. The following results are reported as “mean (lower bound of the confidence interval, upper bound of the confidence interval).” (G) The thickness was 92.3 (28.5, 156.2) μm for wild-type mice (n = 3; 1 female, 2 male), 55.8 (35.6, 76.0) μm for *DNIIR*^Tg^ mice (n = 5; 2 female, 3 male), and 84.0 (52.9, 115.1) μm for *DNIIR*^Tg^*;Prg4*^Tg^ mice (n = 3; all 3 male). There were the following statistically significant differences (reported as “mean difference (lower bound of confidence interval, upper bound of confidence interval)”): wild-type and *DNIIR*^Tg^ mice (36.5 (8.8, 64.2)) and *DNIIR*^Tg^ and *DNIIR*^Tg^*;Prg4*^Tg^ mice (-28.2 (-55.9, -0.5)).

To assess general histological characteristics of the articular cartilage, H&E staining was performed. Histology confirmed fibrillation in *DNIIR*^Tg^ joints when compared to wild-type controls (Fig 4A, 4B, 4D and 4E). Again, *DNIIR*^Tg^*;Prg4*^Tg^ bitransgenic mice exhibited minimal, if any, fibrillation (Fig 4C and 4F). Furthermore, OA cartilage in general exhibits reduced thickness compared to healthy cartilage [31], so articular cartilage thickness was assessed histologically (Fig 4D–4F, double arrows) and through indentation testing (Fig 4G). As expected, *DNIIR*^Tg^ articular cartilage exhibited reduced thickness compared to wild-type controls. Articular cartilage from *DNIIR*^Tg^*;Prg4*^Tg^ mice exhibited similar thicknesses to the wild-type control suggesting that expression of Prg4 prevented cartilage thinning in the *DNIIR*^Tg^ mice.

**Fig 5:**
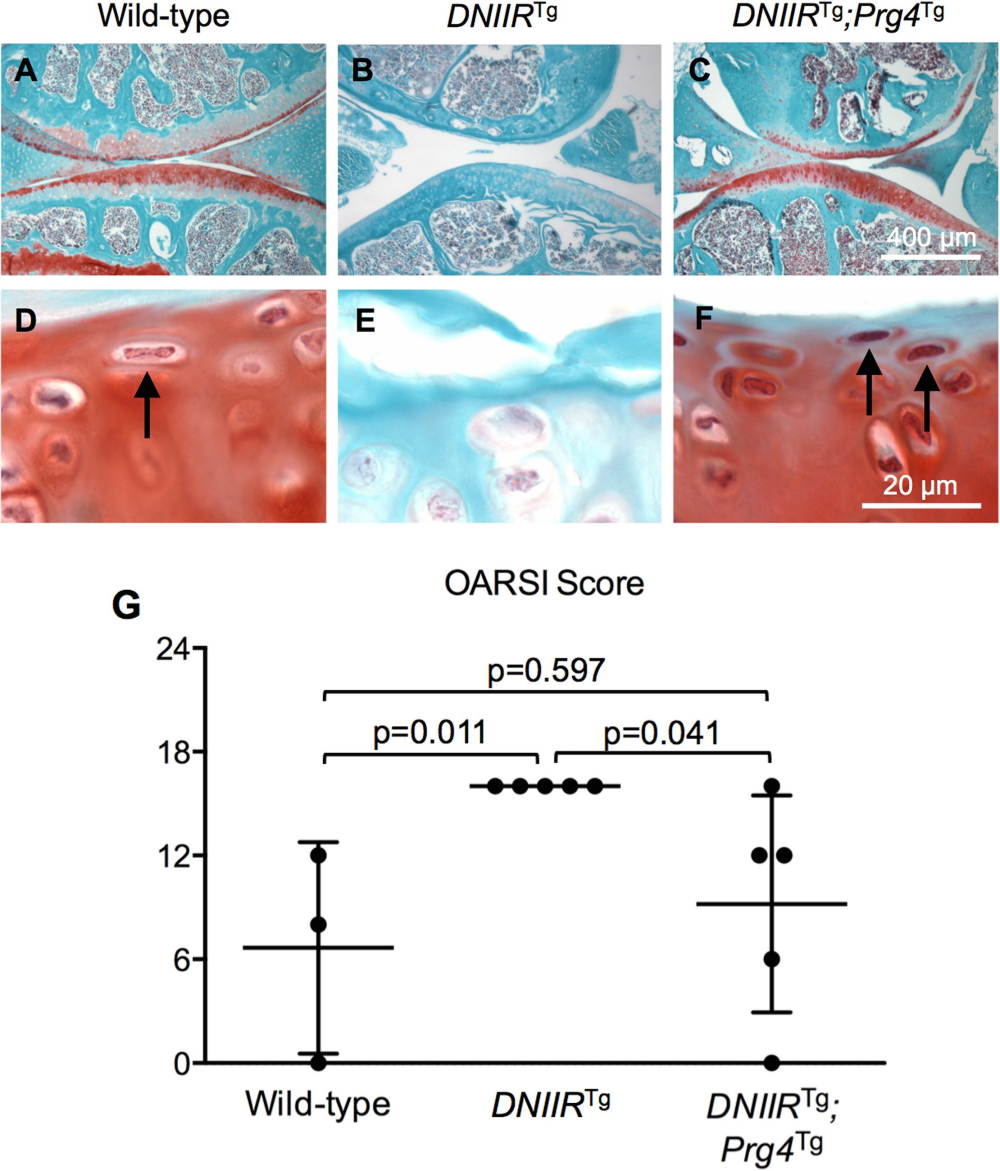
Prg4 prevents loss of proteoglycans and loss of superficial zone cartilage. Safranin O was used to stain for proteoglycans in the articular cartilage of 18-month-old mice. Also, changes in the superficial zone morphology were observed between groups. (A-B, D-E) Wild-type cartilage exhibited a normal superficial zone with cells aligned parallel to the articular surface (arrows point to aligned cells), whereas *DNIIR*^Tg^ cartilage exhibited large regions with no superficial zone as evidenced by a lack of cells aligned parallel to the articular surface. (C and F) *DNIIR*^Tg^*;Prg4*^Tg^ cartilage exhibited a normal superficial zone with cells aligned parallel to the articular surface, similar to the wild-type cartilage. OARSI scoring was performed by an observer blinded to genotype. The following results are reported as “mean (lower bound of the confidence interval, upper bound of the confidence interval).” (G) The OARSI score was 6.7 (-8.5, 21.9) for wild-type mice (n = 3), 16.0 (16.0, 16.0) for *DNIIR*^Tg^ mice (n = 5), and 9.2 (1.4, 17.0) for *DNIIR*^Tg^*;Prg4*^Tg^ mice (n = 5). There were the following statistically significant differences (reported as “mean difference” (lower bound of confidence interval, upper bound of confidence interval)): wild-type and *DNIIR*^Tg^ mice (-9.3 (-18.2, -0.5)) and *DNIIR*^Tg^ and *DNIIR*^Tg^*;Prg4*^Tg^ mice (6.8 (-0.9, 14.5)).

We previously showed that interference of TGF-ß signaling results in reduced Safranin O staining for proteoglycans in articular cartilage [5]. Others previously showed that overexpression of Prg4 in articular cartilage results in increased Safranin O staining [13]. In the present study, cartilage sections were stained with Safranin O. *DNIIR*^Tg^ mice exhibited less Safranin O staining compared to wild-type mice, while *DNIIR*^Tg^*;Prg4*^Tg^ mice exhibited a similar amount of Safranin O staining compared to wild-type mice (Fig 5A–5F) suggesting that Prg4 prevented loss of proteoglycans due to interference of TGF-ß signaling.

Another hallmark of OA in humans is delamination of superficial zone cartilage [27]. The superficial zone is characterized by chondrocytes that are elongated parallel to the articular cartilage surface [27]. *DNIIR*^Tg^ mice exhibited fewer elongated cells compared to wild-type mice (Fig 5D and 5E, arrow); however, when Prg4 was expressed in *DNIIR*^Tg^ mice, more elongated cells at the edge of the articular cartilage were apparent, and this was similar to wild-type controls (Fig 5F, arrows). Delamination of the superficial zone along with fibrillation and proteoglycan staining are major components of OARSI scoring for OA in mice. OARSI scoring was performed on tibial cartilage to quantify histologic changes and OA (Fig 5G). All the *DNIIR*^Tg^ mice that were scored exhibited delamination of superficial zone cartilage across more than 50% of the articular surface, resulting in OARSI scores of 16. Wild-type and *DNIIR*^Tg^*;Prg4*^Tg^ cartilage was intact, resulting in lower OARSI scores. Overall, expression of Prg4 in *DNIIR*^Tg^ mice prevented development of multiple DNIIR-associated OA symptoms.

### Prg4 does not stimulate TGF-β signaling in *DNIIR*^Tg^ mice

**Fig 6:**
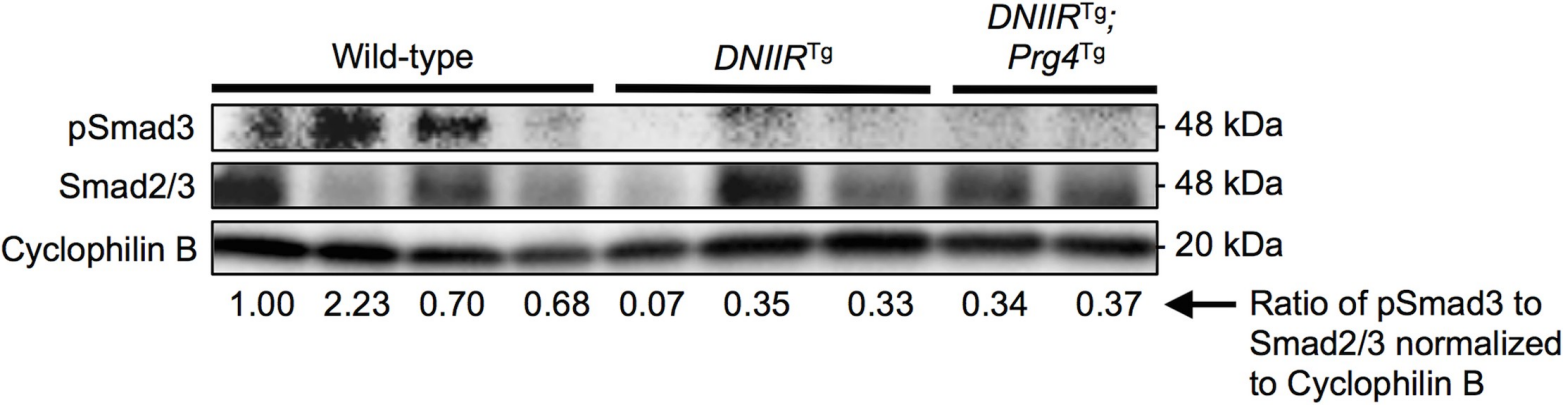
Prg4 does not affect pSmad3 protein levels. pSmad3 and total Smad2/3 protein levels were determined by Western blot. *DNIIR*^Tg^ and *DNIIR*^Tg^*;Prg4*^Tg^ cartilage both exhibited reduced pSmad3 protein levels compared to wild-type cartilage.

Finally, we tested whether Prg4 simply maintained TGF-ß signaling to prevent OA progression. pSmad3 and total Smad2/3, protein levels were assessed via Western blot (Fig 6 and S5–S7 Figs). pSmad3 is indicative of TGF-ß signaling through a well-characterized canonical signaling pathway. As expected, pSmad3 levels were reduced in *DNIIR*^Tg^ cartilage. Maintenance of Prg4 in the *DNIIR*^Tg^ mice did not prevent reduced pSmad3 levels. The results suggest that Prg4 does not simply act by up-regulating TGF-ß signaling.

## Discussion

OA patients with severe joint pain have an increased risk of falling [32–34]. The main cause of falls is stumbling [26]. Behavior of mice with OA has been assessed with a range of behavioral tests [13, 35, 36]. However, previous tests have not measured whether mice with OA stumble while walking. For these reasons, we used an Automatic Foot Misplacement Apparatus to measure the number of steps that mice missed while walking. Our results showed that the apparatus could quantify a stumbling-like behavior. Specifically, *DNIIR*^Tg^ mice missed more steps than all the other mice, confirming that the *DNIIR*^Tg^ mouse model mimics OA behavior. Maintenance of Prg4 expression in the cartilage alleviated this behavior.

Patients with knee OA exhibit fibrillation and thinning of articular cartilage which can progress until subchondral bone is exposed, resulting in pain that prevents normal activity [37, 38]. Therefore, we next tested the hypothesis that maintenance of Prg4 in *DNIIR*^Tg^ cartilage prevented fibrillation and thinning of the cartilage, which might be expected to result in pain and stumbling behavior. Histology and μCT indicated that continued expression of Prg4 in *DNIIR*^Tg^ joints prevented degeneration of the superficial zone and fibrillation of the articular cartilage in addition to maintaining cartilage thickness and proteoglycan content.

While prevention of OA in DNIIR mice is likely due to Prg4’s lubrication functions, we had to rule out the formal possibility that Prg4 might simply stimulate TGF-ß signaling through some unknown mechanism. We then tested the hypothesis by comparing pSmad3 levels in control, *DNIIR*^Tg^ and *DNIIR*^Tg^*;Prg4*^Tg^ mice. Smad3 is the main signaling component of the canonical TGF-β signaling pathway. Maintenance of Prg4 did not keep pSmad3 levels up suggesting Prg4 acted through its lubrication functions. A potential drawback to a PRG4-based treatment that does not completely restore TGF-β signaling is that it may result in additional pathologic effects due to loss of other down-stream targets of TGF-ß, for example 3’-phosphoadenosine 5’-phosphosulfate synthase 2 (*Papss2*) or parathyroid hormone-like hormone (*Pthlh*) [6]. If this is true, restoration of multiple targets of TGF-β signaling together with *PRG4* may be an improvement over treatment strategies focused on *PRG4* alone. However, further work needs to be performed to test these ideas. Also, the *DNIIR*^Tg^*;Prg4*^Tg^ mice did not exhibit osteophytes or synovial hyperplasia. In previous studies, mice treated with TGF-β ligand or adenovirus encoding for TGF-β ligand exhibited increased amounts of proteoglycans in their cartilage but also exhibited osteophytes and synovial hyperplasia [39, 40]. The present study suggests that targeting molecules downstream of TGF-β and SMAD3, such as PRG4, may circumvent osteophyte formation and synovial hyperplasia while still providing the chondroprotective effects of TGF-β. Future work could focus on whether PRG4 affects non-TGF-β signaling pathways, including inflammatory [41, 42] and apoptotic [11, 43] pathways. *DNIIR*^Tg^ mice did not exhibit inflammation in their joints, so the results of the present study are likely not due to changes in inflammation.

It was previously shown that recombinant human PRG4 protected minipigs from post-traumatic OA induced by destabilization of the medial meniscus (DMM) [44]. Post-traumatic OA and OA induced by interference of TGF-ß signaling are very different models. A microarray study compared gene expression between mice subjected to DMM and wild-type mice [45]. When compared to wild-type mice, the articular cartilage of the DMM mice did not exhibit altered expression of *Tgfbr2*, *Pthlh*, and two genes encoding for matrix-modifying enzymes known as *Papss2* and procollagen lysine 2-oxoglutarate 5-dioxygenase 2 (*Plod2*). This is different than the *DNIIR*^Tg^ mouse model, which exhibited downregulation of *Pthlh*, *Papss2*, and *Plod2* [6]. The results suggest that Prg4 may prevent multiple types of OA regardless of the etiology.

There are limitations to the present study. For example, the mice were on combined genetic backgrounds (i.e., C57BL/6 and FVB/N). Little is known about this combination. Mice with C57BL/6-only genetic backgrounds may exhibit lower bone density, altered calcium absorption, and altered sensitivity to OA compared to other backgrounds [46, 47]. Mice with FVB/N-only genetic backgrounds may spontaneously exhibit OA [13].

The present study assessed whether maintenance of Prg4 expression could prevent development of OA associated with interference of TGF-ß signaling in mice. Maintenance of Prg4 prevented foot misplacement, cartilage fibrillation, cartilage thinning, loss of superficial zone cartilage, loss of Safranin O staining, and development of pathologic OARSI scores. Nevertheless, Prg4 did not function by simply stimulating TGF-ß signaling. We propose that Prg4 maintains lubrication in the joint that would normally be lost under conditions of TGF-ß interference thus attenuating OA.

## Supporting information

S1 Fig Prg4 immunohistochemistry control. (A) Histologic sections were stained with primary Prg4 antibody and secondary antibody. (B) Separate histologic sections were stained with only secondary antibody. Without the primary antibody, no staining was visible. A and B show an example from a wild-type mouse.
(TIF)

S2 Fig DNIIR protein expression in mice. DNIIR protein expression was assessed via Western blot. Some mice exhibited low DNIIR protein expression (red arrows), while others exhibited high DNIIR protein expression (all other lanes in *DNIIR*^Tg^ and *DNIIR*^Tg^*;Prg4*^Tg^ groups).
(TIF)

S3 Fig Original uncropped Western blot image shown in S2 Fig. Truncated TGFβR2 bands appeared at approximately 34 kDa. The red box highlights the bands shown in S2 Fig.
(TIF)

S4 Fig Original uncropped Western blot image shown in Fig 1A. Truncated TGFβR2 bands appeared at approximately 34 kDa. The red box highlights the bands shown in Fig 1A.
(TIF)

S5 Fig Original uncropped Western blot image showing representative Cyclophilin B loading control. Cyclophilin B bands appeared at approximately 20 kDa in Figs 1 and 6 and S2 Fig. The red box highlights the bands of interest.
(TIF)

S6 Fig Original uncropped Western blot image shown in Fig 6A. pSmad3 bands appeared at approximately 48 kDa. The red box highlights the location of the pSmad3 bands of interest.
(TIF)

S7 Fig Original uncropped Western blot image shown in Fig 6A. Smad2/3 bands appeared at approximately 48 kDa. The red box highlights the Smad2/3 bands of interest.
(TIF)
