# Supplementary figures and images for: Prg4 prevents osteoarthritis induced by dominant-negative interference of TGF-ß signaling in mice
Source: PLoS One. 2019 Jan 10;14(1):e0210601. doi: 10.1371/journal.pone.0210601 (PMC6328116; doi:10.1371/journal.pone.0210601)

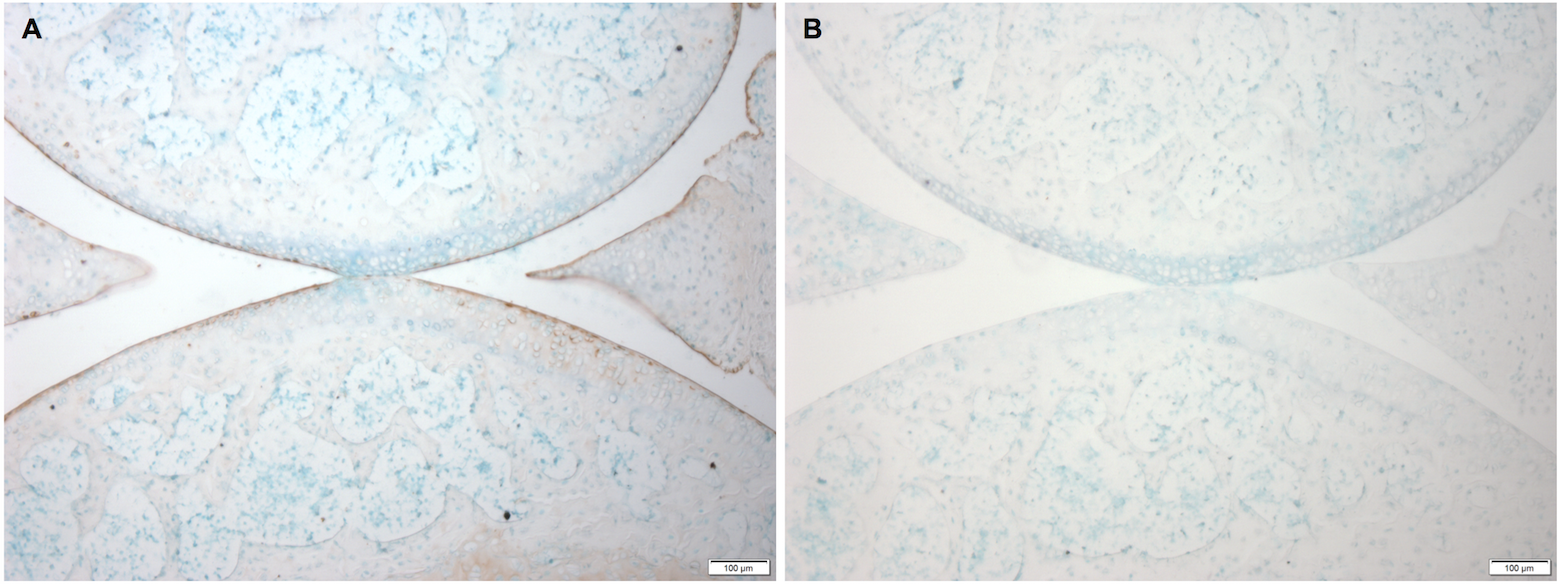

Supplement: S1 Fig — (A) Histologic sections were stained with primary Prg4 antibody and secondary antibody. (B) Separate histologic sections were stained with only secondary antibody. Without the primary antibody, no staining was visible. A and B show an example from a wild-type mouse. (TIF) [file pone.0210601.s001.tif]

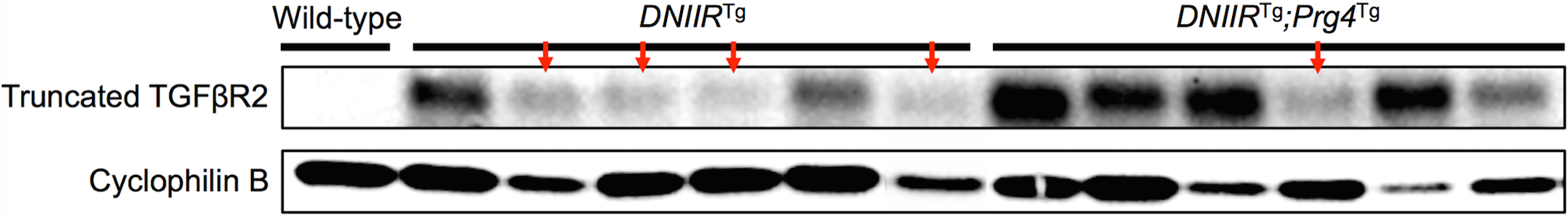

Supplement: S2 Fig — DNIIR protein expression was assessed via Western blot. Some mice exhibited low DNIIR protein expression (red arrows), while others exhibited high DNIIR protein expression (all other lanes in DNIIRTg and DNIIRTg;Prg4Tg groups). (TIF) [file pone.0210601.s002.tif]

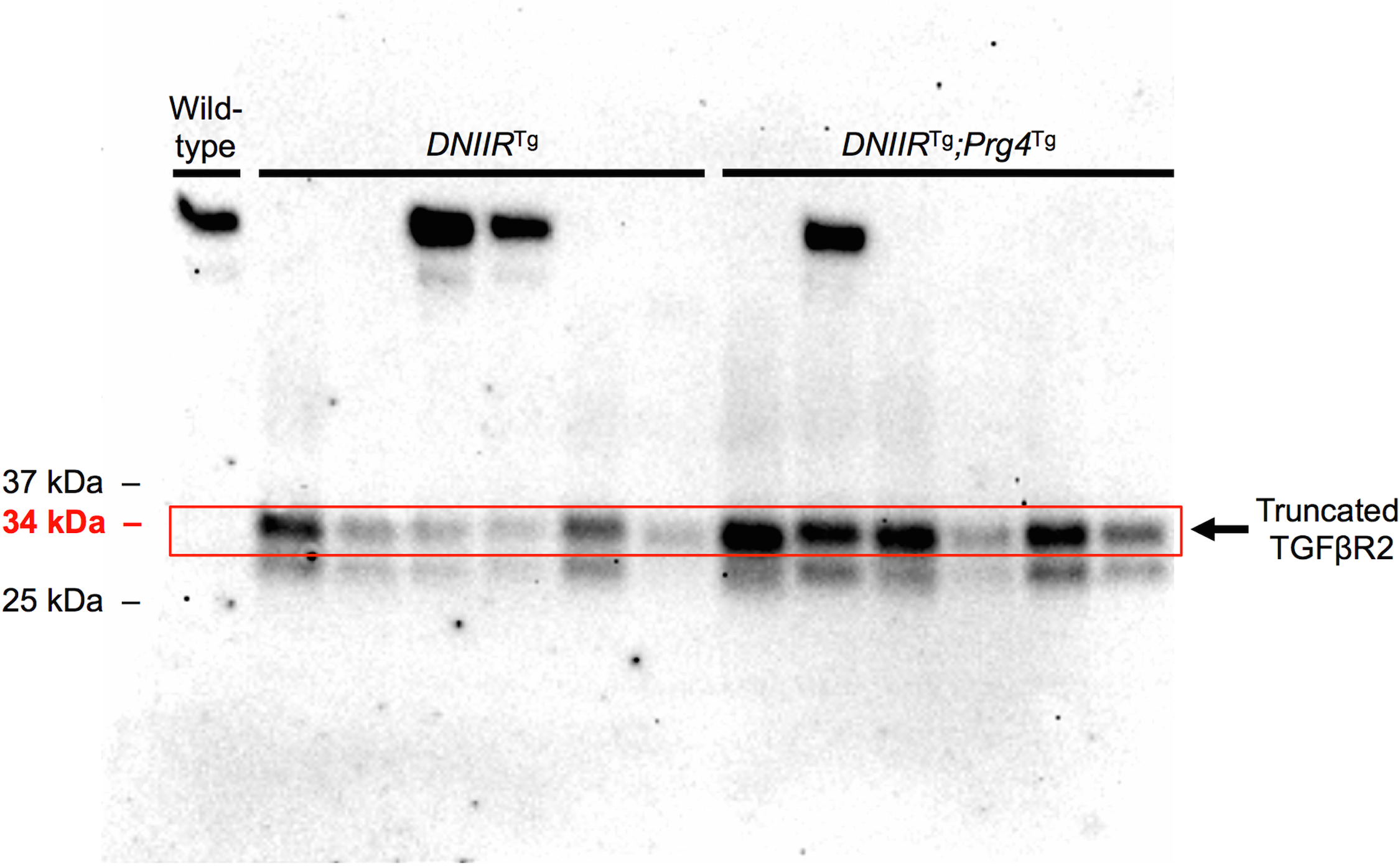

Supplement: S3 Fig — Truncated TGFβR2 bands appeared at approximately 34 kDa. The red box highlights the bands shown in S2 Fig. (TIF) [file pone.0210601.s003.tif]

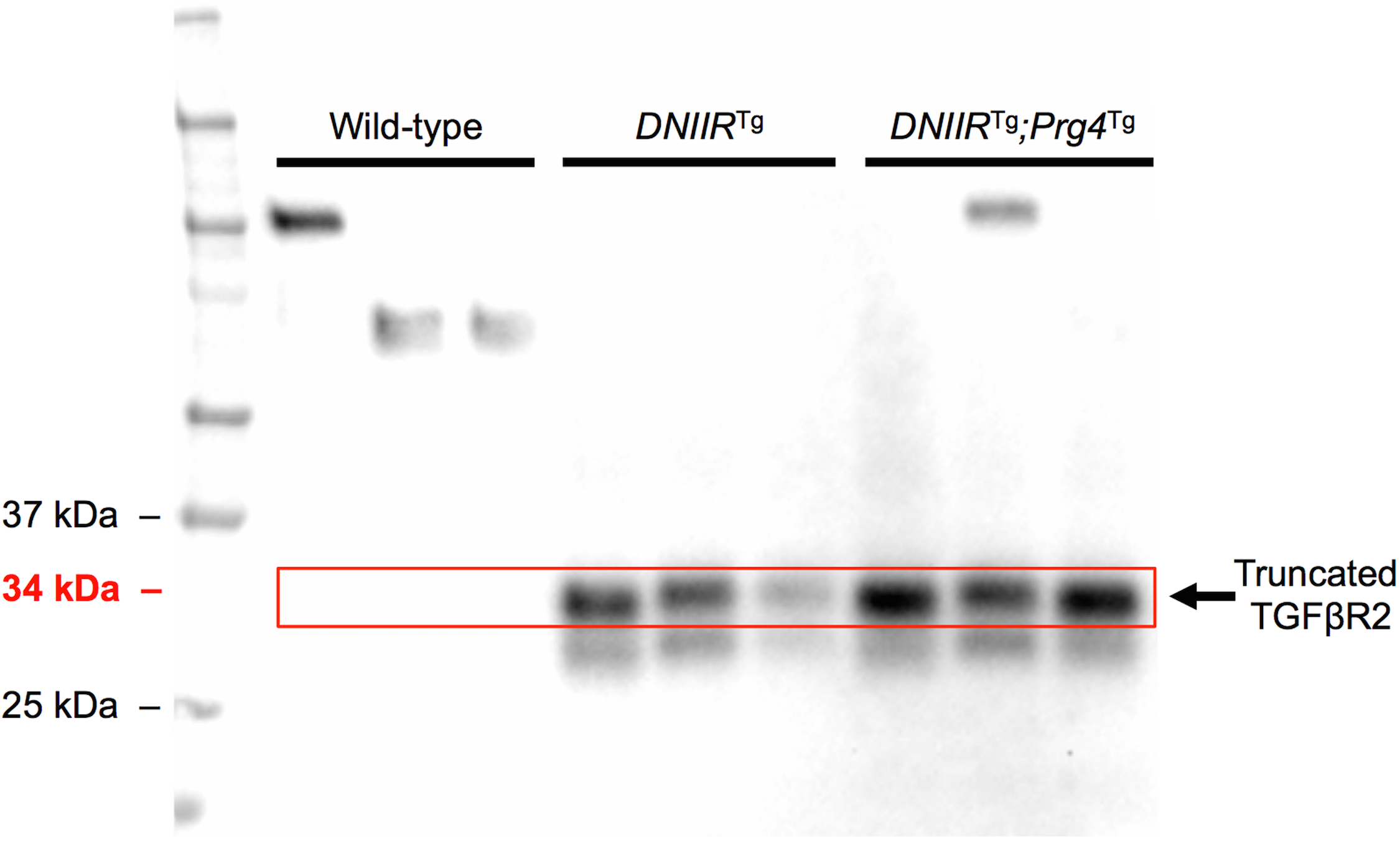

Supplement: S4 Fig — Truncated TGFβR2 bands appeared at approximately 34 kDa. The red box highlights the bands shown in Fig 1A. (TIF) [file pone.0210601.s004.tif]

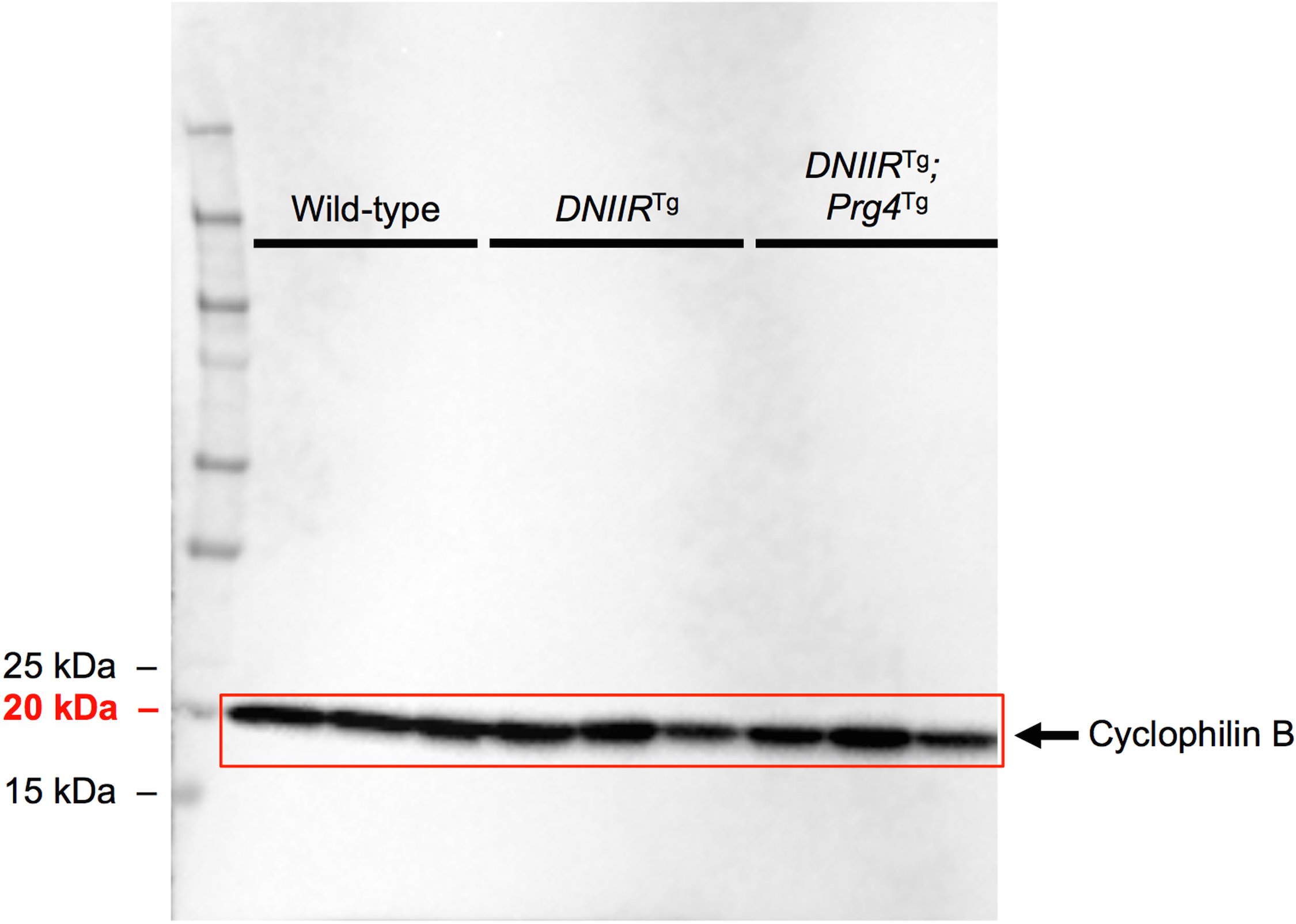

Supplement: S5 Fig — Cyclophilin B bands appeared at approximately 20 kDa in Figs 1 and 6 and S2 Fig. The red box highlights the bands of interest. (TIF) [file pone.0210601.s005.tif]

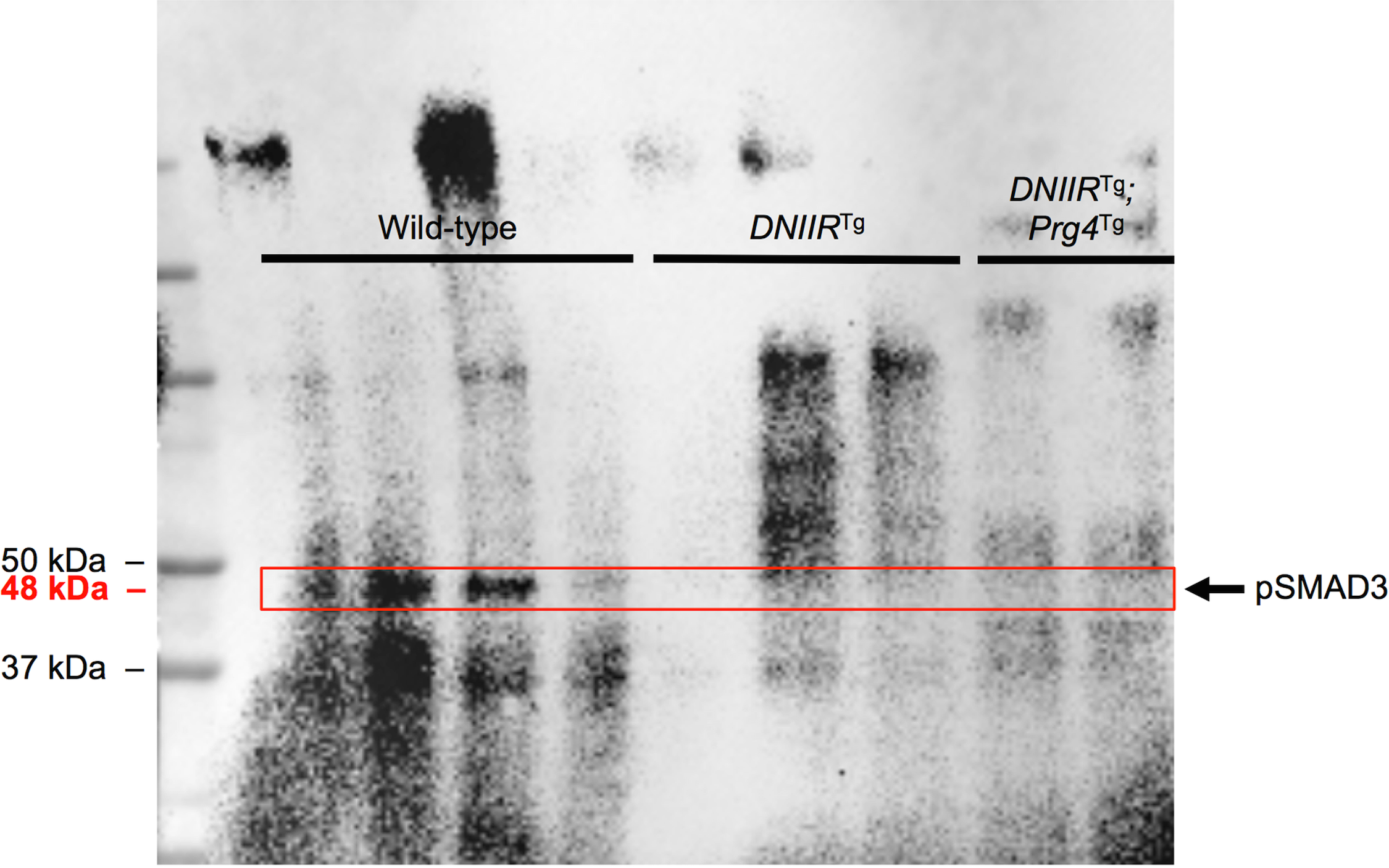

Supplement: S6 Fig — pSmad3 bands appeared at approximately 48 kDa. The red box highlights the location of the pSmad3 bands of interest. (TIF) [file pone.0210601.s006.tif]

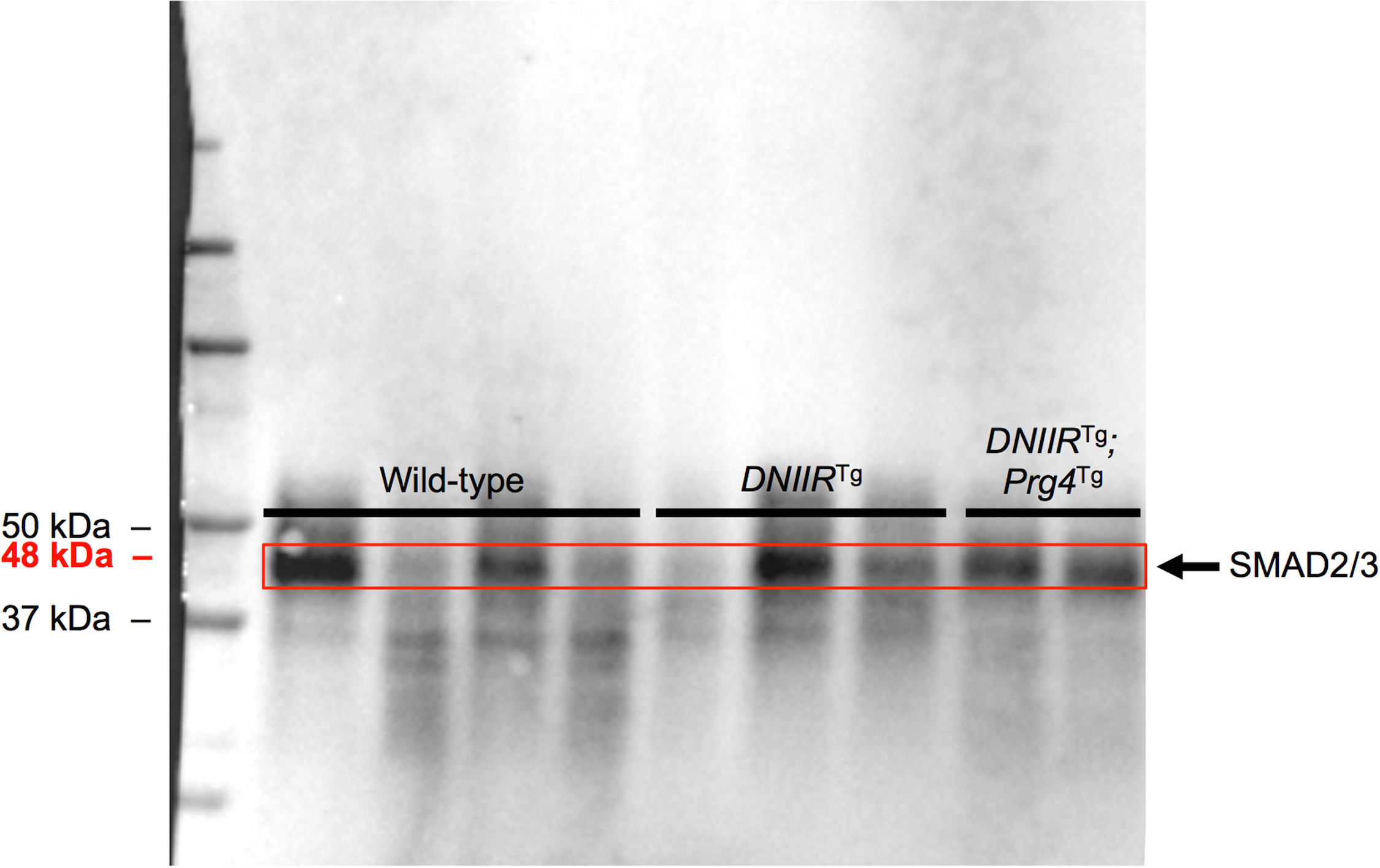

Supplement: S7 Fig — Smad2/3 bands appeared at approximately 48 kDa. The red box highlights the Smad2/3 bands of interest. (TIF) [file pone.0210601.s007.tif]
